# Optimized summary-statistic-based single-cell eQTL meta-analysis

**DOI:** 10.1038/s41598-025-08808-3

**Published:** 2025-08-04

**Authors:** Maryna Korshevniuk, Harm-Jan Westra, Roy Oelen, Monique G. P. van der Wijst, Lude Franke, Marc Jan Bonder, sc-eQTLGen Consortium, José Alquicira-Hernández, Daniel Kaptijn, Maryna Korshevniuk, Jimmy Tsz Hang Lee, Lieke Michielsen, Drew Neavin, Roy Oelen, Aida Ripoll-Cladellas, Martijn Vochterloo, Yoshinari Ando, Odmaa Bayaraa, Irene van Blokland, Mame M. Dieng, M. Grace Gordon, Hilde E. Groot, Pim van der Harst, Chung-Chau Hon, Youssef Idaghdour, Vinu Manikanda, Jonathan Moody, Martijn C. Nawijn, Yukinori Okada, Oliver Stegle, Woong-Yang Park, Deepa Rajagopalan, Tala Shahin, Jay W. Shin, Gosia Trynka, Harm-Jan Westra, Seyhan Yazar, Jimmie Ye, Martin Hemberg, Ahmed Mahfouz, Marta Melé, Joseph E. Powell, Lude Franke, Monique G. P. van der Wijst, Marc Jan Bonder

**Affiliations:** 1 Department of Genetics, University of Groningen, University Medical Center Groningen, Groningen, The Netherlands; 2 Oncode Institute, Utrecht, The Netherlands; 3 Division of Computational Genomics and Systems Genetics, German Cancer Research Center, Heidelberg, Germany; 4 Genome Biology Unit, European Molecular Biology Laboratory, Heidelberg, Germany; 5 Garvan-Weizmann Centre for Cellular Genomics, Garvan Institute of Medical Research, Sydney, Australia; 6 Computational Genomics, Institute for Molecular Bioscience, University of Queensland, Brisbane, Australia; 7 Oncode Institute, Utrecht, The Netherlands; 8 Wellcome Sanger Institute, Wellcome Genome Campus, Cambridge, UK; 9 Department of Human Genetics, Leiden University Medical Center, Leiden, The Netherlands; 10 Leiden Computational Biology Center, Leiden University Medical Center, Leiden, The Netherlands; 11 Delft Bioinformatics Laboratory, Delft University of Technology, Delft, The Netherlands; 12 Life Sciences Department, Barcelona Supercomputing Center, Barcelona, Catalonia Spain; 13 Laboratory for Genome Information Analysis, RIKEN Center for Integrative Medical Sciences, Yokohama, Japan; 14 Biology Program, New York University Abu Dhabi, Abu Dhabi, United Arab Emirates; 15 Department of Cardiology, University of Groningen, University Medical Center Groningen, Groningen, The Netherlands; 16 Biological and Medical Informatics Graduate Program, University of California San Francisco, San Francisco, USA; 17 UCSF Division of Rheumatology, Department of Medicine, University of California San Francisco, San Francisco, CA USA; 18 Institute for Human Genetics, University of California San Francisco, San Francisco, USA; 19 Department of Bioengineering and Therapeutic Sciences, University of California San Francisco, San Francisco, USA; 20 Department of Cardiology, University Medical Center Utrecht, Utrecht, the Netherlands; 21 Department of Pathology and Medical Biology, University of Groningen, University Medical Center Groningen, Groningen, the Netherlands; 22 GRIAC Research Institute, University Medical Center Groningen, Groningen, the Netherlands; 23 Department of Statistical Genetics, Osaka University Graduate School of Medicine, Suita, Japan; 24 Laboratory for Systems Genetics, RIKEN Center for Integrative Medical Sciences, Yokohama, Japan; 25 Samsung Genome Institute, Samsung Medical Center, Seoul, Korea; 26 A*STAR Genome Institute of Singapore, Singapore, Singapore; 27 Open Targets, Wellcome Genome Campus, Cambridge, UK; 28 Bakar Computational Health Sciences Institute, University of California San Francisco, San Francisco, CA USA; 29 Department of Epidemiology and Biostatistics, University of California San Francisco, San Francisco, CA USA; 30 Parker Institute for Cancer Immunotherapy, San Francisco, CA USA; 31 Chan Zuckerberg Biohub, San Francisco, CA USA; 32 Evergrande Center for Immunologic Disease, Harvard Medical School and Brigham and Women’s Hospital, Boston, USA; 33 UNSW Cellular Genomics Futures Institute, University of New South Wales, Sydney, Australia

**Keywords:** eQTL, Weighted meta-analysis, scRNA-seq, Functional genomics, Quantitative trait

## Abstract

The identification of expression quantitative trait loci (eQTLs) holds great potential to improve the interpretation of disease-associated genetic variation. As many such disease-associated variants act in a context-, tissue- or even cell-type-specific manner, single-cell RNA-sequencing (scRNA-seq) data is uniquely suitable for identifying the specific cell type or context in which these genetic variants act. However, due to the limited sample sizes in single-cell studies, discovery of cell-type-specific eQTLs is now limited. To improve power to detect such eQTLs, large-scale joint analyses are needed. These are however, complicated by privacy constraints due to sharing of genotype data and the measurement and technical variety across different scRNA-seq datasets as a result of differences in mRNA capture efficiency, experimental protocols, and sequencing strategies. A solution to these issues is a federated weighted meta-analysis (WMA) approach in which summary statistics are integrated using dataset-specific weights. Here, we compare different strategies and provide best practice recommendations for eQTL WMA across scRNA-seq datasets.

## Introduction

As the scale, cost per cell and sensitivity^1–3^ of single-cell RNA-sequencing (scRNA-seq) have improved significantly over the last decade, scRNA-seq has grown in popularity as a potent alternative to bulk RNA-seq^4^. These developments have paved the way for the generation of large numbers of population-based scRNA-seq datasets^5^, which often also include both genetic and donor-specific information. Such datasets are a valuable resource for studying the effects of genetic variation on the expression of genes in individual cell types through expression quantitative trait locus (eQTL) mapping^6^.

In large-scale multi-study eQTL mapping efforts, data analysis is generally performed in one of two ways. The first approach is a single centralized eQTL mapping across different datasets, which is possible when direct access to all data is available. In this kind of “mega-analysis”^7,8^, privacy-sensitive data (such as genetic information) is shared and stored at one centralized site. The second approach is to perform a federated or meta-analysis, for instance by combining summary statistics from multiple independently analyzed datasets. In a meta-analysis based on summary statistics, there is no need to share privacy-sensitive data, and the only procedure that needs to be centralized is the integration of summary statistics. This also means that new datasets can be easily added to the analysis without having to reprocess all the previously included datasets. Taken together, the meta-analysis approach has several benefits that make it an often-used choice in the context of large (inter)national efforts that are continuously expanding.

There are several approaches for combining summary statistics from multiple cohorts through meta-analysis, based on e.g.,* p*-values, Z-scores, betas, or standard errors. One of the most-used methods is Fisher’s method (Methods)^9–12^. According to Whitlock and Zaykin^9^, the weighted Z-score, or weighted meta-analysis (WMA), outperforms its alternatives (Stouffer’s^13^, Lipták’s^14,15^, etc.) in terms of type I error and power when integrating summary statistics. In studies based on bulk RNA-seq, a widely applied strategy for combining eQTL summary statistics is to use the square root of the cohort sample size (a cohort-specific weight), for example as it is implemented in the METAL meta-analysis tool^10^, or the inverse square of the standard error of the eQTL effect (an eQTL-specific weight)^9^.

While these approaches have been useful for large-scale bulk RNA-seq studies, single-cell studies are currently generally smaller in size and show more variability in quality-related parameters compared to bulk-based studies. Smaller size of single-cell datasets indicates the need of using fixed-effect meta-analysis^16^ and an importance of identifying the most effective meta-analysis weights for such datasets. Important factors to take into consideration when meta-analyzing scRNA-seq datasets are, for example, the scRNA-seq technology and version chemistry applied (e.g. Smart-seq2^17^, Chromium 10X Genomics version 2 and version 3 (10X V2 and V3^18,19^)), as these can have widely differing characteristics. For instance, a typical 10X Chromium experiment quantifies about 10 times fewer genes, but 100 times more cells, than a typical Smart-seq2 experiment. These differences can result in a different signal-to-noise ratio per gene for Smart-seq2 approaches compared to Chromium assays. Knowing the best way to integrate such datasets in a meta-analysis context will be especially relevant for large consortia such as the Human Cell Atlas^20^ or single-cell eQTLGen^5^, which both aim to combine signals from thousands of donors and many different datasets.

In this study, we aimed to identify the optimal strategy for meta-analyzing the output of multiple single-cell datasets, with a specific emphasis on eQTLs. We employ a WMA of (single-cell) eQTL summary statistics and benchmark different gene- and dataset-specific characteristics as weights, with the goal of improving upon classical sample-size or standard-error WMA approaches.

## Results

### WMA of single-cell RNA-seq eQTL data

**Fig. 1:**
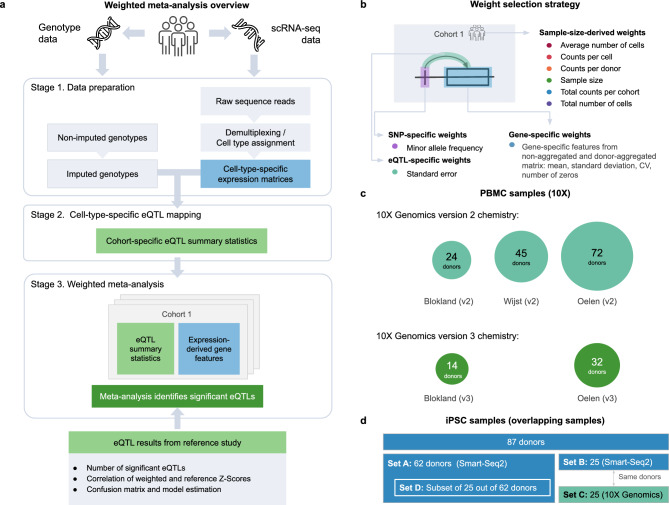
Workflow and weighting strategies for meta-analysis of single-cell eQTL data. (**a**) The general workflow for weighted meta-analysis. (**b**) The cohort-, gene-, SNP-, and eQTL-specific weights used in the meta-analysis. (**c**, **d**) Description of the (**c**) peripheral blood mononuclear cell (PBMC) and (**d**) induced pluripotent stem cell (iPSC) datasets used in the analysis. The PBMC data consists of five single-cell datasets (n = 187 donors: 141 with 10X V2 and 46 with 10X V3 chemistry). The iPSC data consists of two single-cell datasets (n = 87 donors: 87 Smart-Seq2 and 25 donor-matched 10X V2). To analyze independent sample sets and test for the effects of combining smaller datasets, we split the 87 Smart-Seq2 samples into Set A (62 non-10X-matched samples) and Set C (25 10X-matched samples). Set A was further split into a smaller set of 25 non-10X-matched samples (Set D).

To systematically analyze how to best meta-analyze scRNA-seq-derived eQTL mapping summary statistics, we first generated dataset-specific eQTL summary statistics and tested different meta-analysis weights for each used dataset separately (Fig. 1a, roup of donor-mb, Methods). For this, we used two groups of datasets: 1. a group of five human peripheral blood mononuclear cells (PBMC) 10X V2 or V3 chemistry datasets (Fig. 1c; Supplementary Table 1a-b, Supplementary Figure 1 and 2). A group of donor-matched induced pluripotent stem cell (iPSC) datasets generated using either 10X or Smart-Seq2 technology (Fig. 1d; Supplementary Table 1c, Supplementary Figure 2). This first group of PBMC datasets mainly assesses meta-analysis results within a single technology platform, whereas the second group of iPSC datasets assesses meta-analysis results across different technology platforms. Together, both analysis groups provide some insight in the generalizability of our results.

As the starting point of our study, we used the well-established WMA weights: sample size (i.e. number of donors) and standard error. We additionally focused on using dataset-, SNP- and/or gene-specific characteristics to improve over the sample size based weighting in meta-analysis, as these statistics are often available, while sharing standard error information is more challenging due to the significant increase of the data sharing. To leverage the nature of single cell data and aid power in WMA for single cell studies, we assessed the impact of single-cell-derived summary statistics and introduced single cell specific weights (*1. the average number of cells per donor, 2. the average number of molecules detected per cell, 3. the average number of molecules detected per donor, 4. the total number of cells per cohort, and 5. the total number of molecules detected per cohort*) (Fig. 1b). These single-cell-derived weights may capture parameters that independently drive eQTL discovery power in single-cell data and are only partially captured by bulk-derived weighting for the standard-error or the sample size.

### WMA in the PBMC datasets

As input for the meta-analysis, we utilized cis-eQTL summary statistics obtained through genome-wide cis-eQTL mapping conducted separately in each of the five datasets, using both pseudobulked PBMC and pseudobulked monocyte samples (Fig. 1a**, **Supplementary Fig. 1a–g, Methods). To ensure consistency and comparability across studies, the mapping was restricted to SNPs and genes that were present in all five datasets as well as in the corresponding reference datasets used for benchmarking.

We employed two reference datasets to benchmark our weighting strategy. The first was the eQTLGen dataset, comprising bulk whole-blood eQTL data derived from 31,684 individuals, which served as the benchmarking reference for both PBMC and monocyte data^21^. The second was the OneK1K dataset, which provided monocyte-specific eQTL statistics and was used to evaluate the performance of the monocyte-specific weighted meta-analysis^7^. Using the eQTLGen reference, we retained 607,445 SNPs and 9935 genes for analysis, whereas the OneK1K-based filtering resulted in 522,622 SNPs and 8682 genes.

To identify significant cis-eQTLs, we performed 1,000 gene-level permutations followed by correction using the Benjamini–Hochberg False Discovery Rate (BH FDR), applied to the top eQTL per gene. Associations were considered statistically significant at an FDR threshold of less than 10% (Methods). Overall, we observed a high degree of concordance in the set of significant eQTLs across individual datasets, as well as strong correlation with those identified in the bulk eQTLGen reference dataset (Supplementary Fig. 3a–e).

To determine the most effective strategy for integrating single-cell eQTL summary statistics derived from cohorts using different 10X Genomics chemistries, we focused on pseudobulk PBMC and monocyte sc-eQTL data as input (Fig. 1c). PBMC data was selected for its expected high overlap with whole-blood profiles, while monocyte data enabled assessment of method performance within a well-characterized and consistently represented single cell type.

Using the pseudobulk PBMC sc-eQTL results derived from the five datasets, we conducted 11 different meta-analyses: one meta-analysis of all five datasets together and 10 pairwise analyses of the individual combinations. To estimate the performance of different WMA strategies, we focused on the number of unique genes with at least one significant *cis*-eQTL effect, which we call ‘eGenes’. Subsequently, we determined how many of these eQTLs were replicated using the F1* score (which ranges from 0–1 (poor-good), an adaptation of the F1 score that considers the large power discordance between bulk and single-cell studies (see Methods). We defined the F1*, by only considering an eQTL as true positive if it was identified in the reference dataset and at least one sc-eQTL WMA. The F1* metric takes into account precision and recall, providing a balanced measure to determine whether a specific meta-analysis strategy correctly identifies true positive eQTLs while concurrently minimizing both false positives and negatives.

**Fig. 2:**
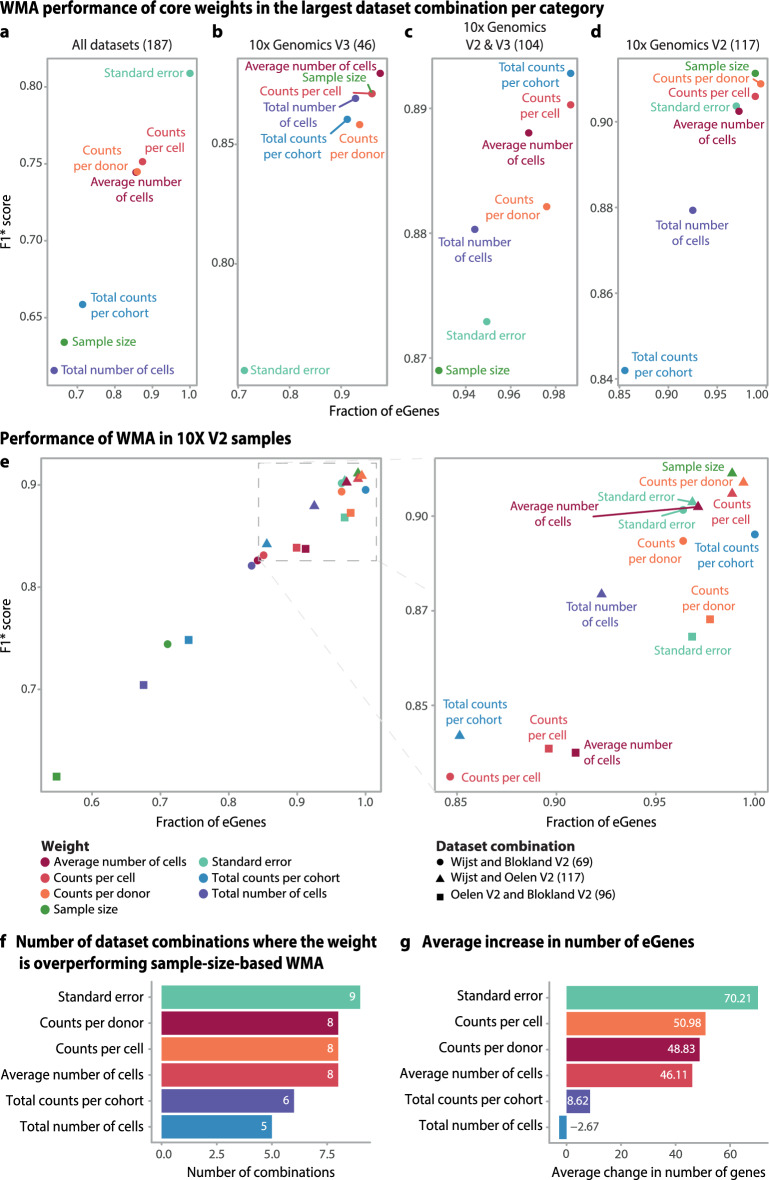
Results of the improved sc-eQTL weighted meta-analysis in the PBMC pseudobulk datasets: (**a**–**d**) Comparison of the fraction of eGenes detected and accuracy (F1* score) in WMA over all five datasets (**a**), 10X V3 chemistry (**b**), 10X V2 and V3 chemistries (**c**), and 10X V2 chemistry combinations (**d**). **e** Three pairwise 10X V2 chemistry dataset combinations. The expansion at right enlarges the top-performing weights in these comparisons. (**f**, **g**) Comparison of the best-performing weights among 10 pairwise dataset combinations in terms of the (**f**) increase in the number of eGenes and (**g**) the weighted mean of the eGenes change in comparison to sample-size weighting.

**Fig. 3:**
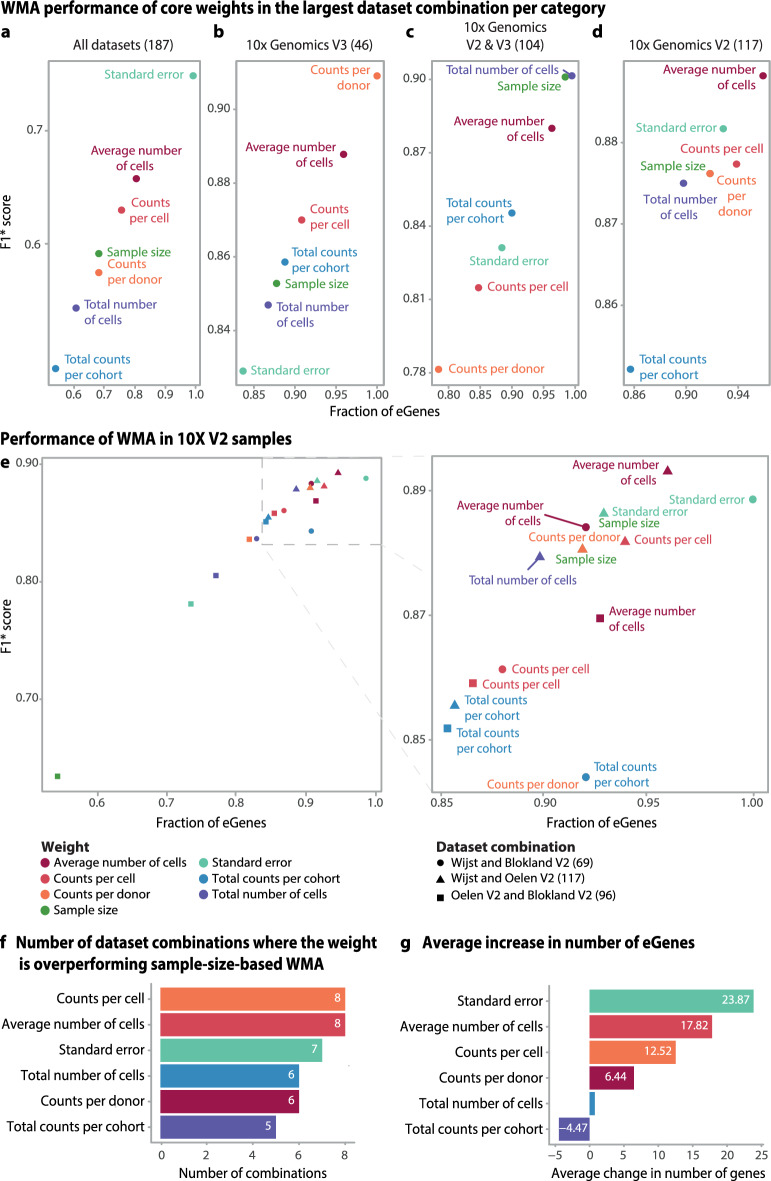
Meta-analysis of sample-size-like weights in monocyte datasets. (**a**–**d**) Combination of the number of eGenes detected and accuracy (F1* score) for (**a**) all five datasets and (**b**) sample-size-wise the largest pairwise comparisons of 10X V3 chemistry, (**c**) 10X V2 and V3 chemistries, and (**d**) 10X V2 chemistry combinations. (**e**) Three pairwise 10X V2 chemistry dataset combinations. The expansion at right enlarges the top-performing weights. (**f**, **g**) Comparison of the (**f**) main weights among 10 pairwise dataset combinations in terms of the increase in the number of eGenes and (**g**) the weighted mean of eGenes change.

Testing different weights, we found that the standard-error-based weighting performed best in the meta-analysis of all five datasets (Figs. 1c, 2a), detecting 212 (50%) more eGenes than the sample-size-based WMA and increasing the F1* score by 0.17. Although this standard error weight also performed well in the pairwise analyses (Fig. 2b–e), other metrics performed better. For example, both *counts per cell* and *average number of cells* were best performing in 8 out of the 10 pairwise meta-analyses (Fig. 2f,g). Compared to sample-size-based weighting, weighting by *counts per cell* improved the number of eGenes identified by 36% on average (51 eGenes) and led to a 0.112 improvement in F1* score, whereas weighting by *average number of cells* improved the number of eGenes by 30% (46 eGenes) and the F1* score by 0.098 (Fig. 3a–d, Supplementary table 2a).

We next tested these WMA strategies in pseudobulked monocytes to determine whether the results of the PBMC analysis are transferable to a single cell type, the resolution at which eQTL mapping in scRNA-seq data is mostly conducted. We selected monocytes for two reasons. First, they show relatively low cell subtype heterogeneity. Second, as one of the major cell types, monocytes provide sufficient statistical power to map and meta-analyze eQTLs. We excluded the Blokland V2 & Blokland V3 dataset combination from this comparison as its monocyte counts were insufficient to detect any eQTLs (Supplementary table 1b). Similar to the PBMC data, we observed that the standard-error-based method for WMA performed best when integrating all sc-eQTL studies but was not optimal for the pairwise combinations (Supplementary table 2b, Fig. 3a–e). We found that, compared to sample-size-like-weighting, using the *average number of cells per donor* improved the number of genes detected in 8 out of 10 dataset combinations in both references (Supplementary figure 4a,b), showing an increase of up to 50% in eGenes (+18) and an increase of 0.126 in F1*, leading to detection of an additional 17 eGenes on average in eQTLGen reference (Fig. 3f,g).

Altogether, the results from pseudobulk PBMC and monocyte samples suggest that incorporating single-cell-specific characteristics as weights in the meta-analysis enhances both the number of detected eGenes and the F1* score. Notably, the top-performing weights in both pseudobulk samples, and for monocytes in both references (eQTLGen and OneK1K) were consistent (*average number of cells per donor* and *counts per cell*).

### WMA of Smart-seq2 and 10X data in iPSCs

While 10X is the most-used scRNA-seq technology for large-scale population-based studies, other technologies such as full-length Smart-seq2 can be more suitable for specific research questions, e.g. those focusing on isoform usage or RNA-velocity. Therefore, we next assessed how to best meta-analyze multiple Smart-seq2 datasets and how to combine full-length Smart-seq2 with 3’-end 10X data (Supplementary figure 2a-f). For this, we leveraged 87 samples from the HipSci study^22^, which we divided into different sets (Fig. 1d). Each of the donors was genotyped and had Smart-Seq2 scRNA-seq data available, with matched 10X V2 scRNA-seq data (set C) available for 25 of the Smart-Seq2 samples (Set B). We split the original scRNA-seq dataset of 87 donors into four sets (as shown in Fig. 1c) to test meta-analysis of 10X and Smart-seq2 datasets (Sets A & C, Sets C & D) or multiple Smart-seq2 datasets (Sets A & B, Sets B & D). As a reference dataset, we used a set of 526 iPSC samples for which bulk RNA-seq and eQTL statistics were available. This set also included all 87 samples for which Smart-seq2 was available^23^.

**Fig. 4:**
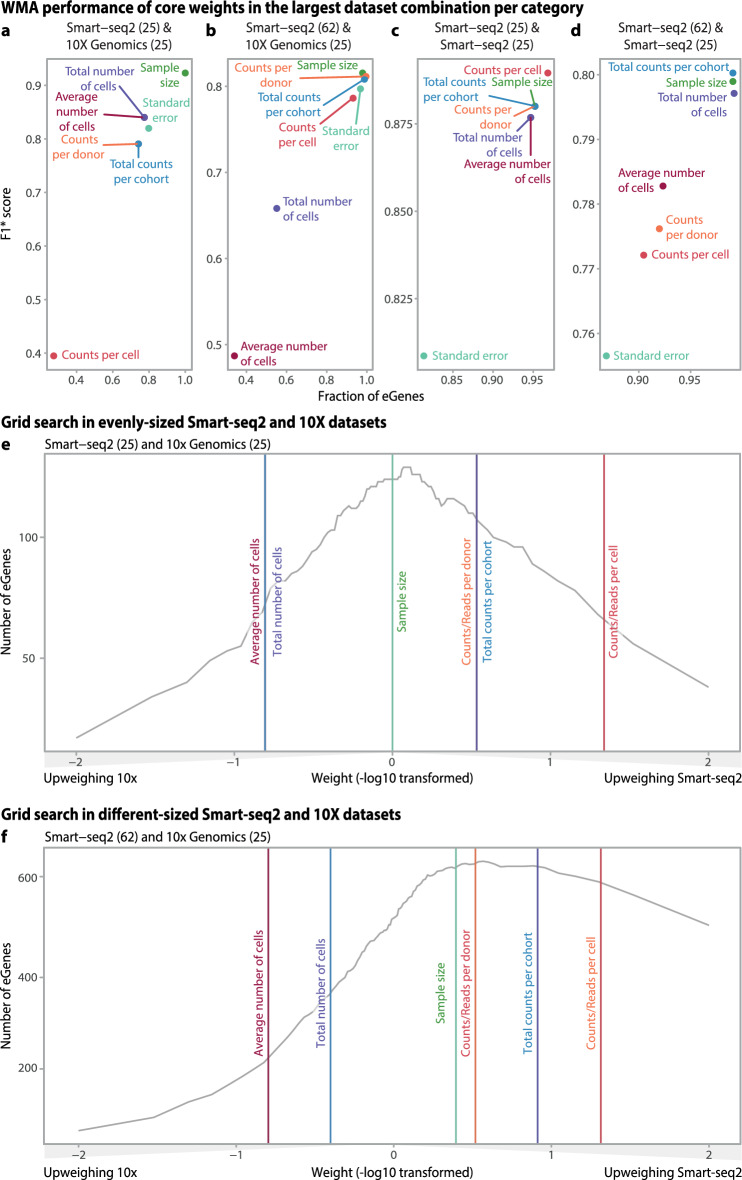
Weighted meta-analysis of sample-size-like characteristics in iPSC samples. **a**–**d** The number of eGenes detected and accuracy (F1* score) for all four iPSC datasets. **e**, **f** Results of the grid search in combinations of Smart-seq2 (Set B or C) and 10X (Set D) samples. Vertical lines indicate the ratio of weight of Set C versus weight of Set D (**e**) or of Set C versus Set D (**f**) for one of the weights of the WMA.

When comparing to other weights (including standard error), we observed that weighting the two evenly sized 10X and Smart-seq2 datasets (Sets B & C, Fig. 1d) equally (i.e. using *sample size*) performed best, leading to detection of 124 eGenes and an F1* score of 0.93 (Fig. 4a**)**. All other weights performed equally and slightly worse (95.75 ± 2.9 eGenes detected and F1* score = 0.816 ± 0.025, Supplementary table 2c), except *counts per cell,* which showed very poor performance (34 eGenes detected and F1* score = 0.395).

When meta-analyzing the two differently sized 10X and Smart-seq2 datasets (Sets C and A, Fig. 1d), *standard error*, *sample size, counts per cell, counts per donor,* and *total counts per cohort* showed similar performances (637 eGenes detected and F1* score = 0.803, Fig. 4b). The worst-performing weights were *average number of cells* (223 eGenes detected and F1* score = 0.49) and *total number of cells* (361 eGenes detected and F1* score = 0.66) (Supplementary table 2c), indicating that accounting for the differences in the number of cell-related parameters does not improve WMA performance.

When combining Smart-seq2 datasets, we observed that different sample-size-like weights perform similarly. In the meta-analysis of evenly sized datasets (Sets B and D, Fig. 1d), all the sample-size-like weights showed similar performance, enabling detection of 178 eGenes and showing an F1* score of 0.88 (Fig. 4c). In contrast, standard-error-based weighting showed suboptimal performance compared to sample-size*-*based weighting, leading to detection of 153 eGenes and an F1* of 0.81 (Supplementary table 2c). In the meta-analysis of differently sized datasets (Sets A and B, Fig. 1d), three parameters performed equally well: *sample size, total number of cells,* and *total counts per cohort* (853 eGenes detected and F1* = 0.80, Fig. 4d). Three other parameters, *average number of cells per donor, counts per donor*, and *counts per cell*, showed worse performance compared to sample-size-based weighting (795 eGenes detected and F1* = 0.78). *Standard error* again showed the worst performance (748 eGenes detected and an F1* score 0.75 lower than sample-size-based weighting).

To determine whether it would be possible to further improve upon the best-performing (i.e. sample-size-like derived) weighting of 10X and Smart-seq2 data, we conducted a systematic grid search (Methods) on the evenly sized Smart-seq2 and 10X sets (Sets D and C, Fig. 1d). We did this by starting from a point where most weight was given to the 10x dataset and then changing the meta-analysis weights in small increments until most weight was given to the Smart-seq2 dataset. This revealed that most eGenes were detected near the point corresponding to the sample size weight (Fig. 4e, Supplementary table 3a).

When combining the larger Smart-seq2 dataset (n = 62) with the 10X dataset (n = 25), we observed that three weighting strategies, *sample size, total counts per cohort*, and *counts per donor*, performed comparably well (646 ± 5.5 eGenes detected and F1* score = 0.812 ± 0.003), while the other approaches yielded worse results (Fig. 4f, Supplementary table 3b).

We see that when further up weighing the Smart-seq2 data we get slightly more eGenes, indicating that the Smart-seq2 data has slightly more power than the 10X dataset. However, none of our previously selected weights matched this optimal weight better than the *sample-size* itself.

Overall, in the meta-analysis of Smart-seq2 samples, we observed a strong similarity in the performance of sample-size-like weights, while the standard-error-based weighting method showed poorer performance (Fig. 4). From the grid-search we find that sample size-based weighting is matching the peak of the WMA performance for both equally- and differently sized combinations of Smart-seq2 and 10X indicating that it is an optimal weight for cross-protocol data meta-analysis.

### Improving WMA by adding a secondary weighting parameter

**Fig. 5:**
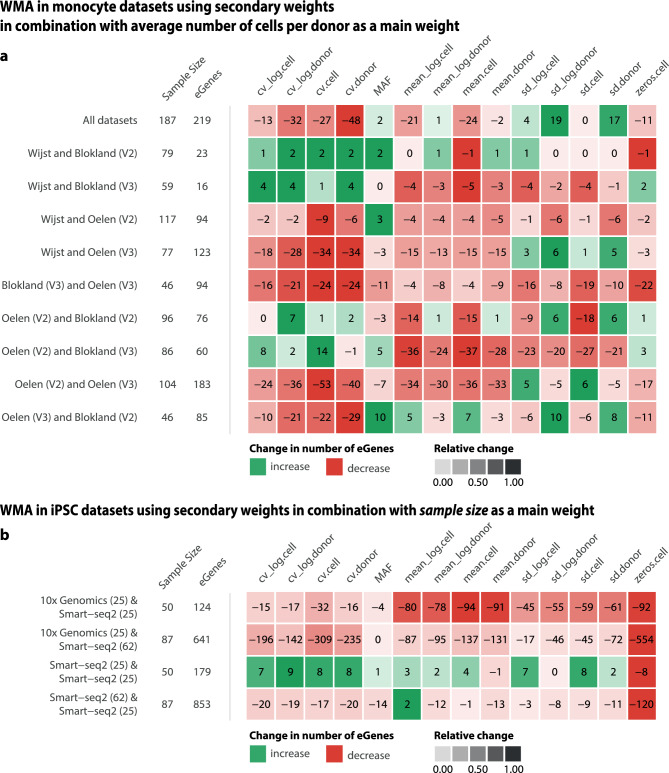
Weighted meta-analysis with secondary weights on **a** monocyte and **b** iPSC datasets employing secondary weights derived from gene expression features and minor allele frequency (MAF) in combination with overall best-performing sample-size-derived weights. **a** For monocyte samples, we used the average number of cells per donor as a main weight. **b** For iPSC samples, we used sample size as a main weight. Numbers in the heatmap indicate the change in the number of eGenes detected. Green and red indicate an increase or decrease in the number of eGenes compared to the main weight, respectively. Color saturation indicates the fold-change of the number of eGenes detected.

As suggested by the grid search (Fig. 4e, f), eQTL discovery may be further improved by adding an additional weight. We therefore conducted a set of WMA in monocytes (Fig. 5a, Supplementary figure 4c) and iPSC samples (Fig. 5b) in which we integrated secondary weights that reflect SNP- or gene-specific information on top of the overall-best performing sample-size-derived weight (i.e. average numbers of cells per donor and sample size for monocytes and iPSC samples, respectively) (Fig. 1b, Supplementary table 4,5). We chose not to do further transformation of the SE based WMA as the SE should already reflect a combination of the now combined parameters, limiting the chance of overfitting.

Testing the combined weights in the monocyte data, we observed the largest improvement in terms of additional eGenes when meta-analyzing all five datasets using the combination of *sd.donor* and *average number of cells per donor* as weights. This resulted in an 8% increase in eGenes detected (an additional 19 eGenes) and a 0.037 increase in F1* score compared to the WMA using only the primary weight (*average number of cells per donor* (Fig. 5a, Supplementary table 4). Overall, in comparison to the standard sample-size-based WMA, 51 additional eGenes (27.5%) were detected on average, and we observed an average improvement of the F1* by 0.104. In the pairwise meta-analyses, the overall absolute increase in number of eGenes was much lower (fewer than 15 new eGenes detected) than for all five datasets combined. Nevertheless, this increase in eGenes was meaningful, producing up to a 25% increase in the number of eGenes. For example, using *coefficient of variation on the donor-level* (*cv.donor*) led to a 280% increase in the eGenes detected (+14 eGenes in comparison to *sample-size-based weighting*) in the Wijst and Blokland V3 data. Additionally, the results of the monocyte dataset comparison were consistent across two independent reference datasets—eQTLGen and the OneK1K study—demonstrating the robustness and reproducibility of the findings.

The secondary weight on average decreased the number of eGenes detected in the iPSC WMAs (Fig. 5b). It was only when we combined two evenly sized Smart-seq2 datasets that observed a minor increase in eGenes detected when using *coefficient of variation*-derived parameters or *standard deviation* of donor-aggregated matrices (*sd.donor*), resulting in a 3.3 ± 1.5% increase in eGenes (8 ± 1 new eGenes) and a 0.02 ± 0.01 increase in F1* score. Interestingly, the only secondary weight to yield a small change in eGene detection was weighting for the MAF.

In 5 out of 10 monocyte dataset combinations and 1 out of 4 iPSC dataset combinations, using MAF as secondary weight increased the number of eGenes detected by 4–18% compared to the main weight alone. All other weights improved eGene detection in at least one combination of monocyte and iPSC samples (by up to 17 eGenes), but the performance was much more variable overall.

While conclusions were limited by dataset size and chemistry variations, our results show that certain secondary weights outperform sample-size-based weighting in the pairwise meta-analysis of dataset generated with the same technology. In particular, MAF and standard deviation of expression overperformed in 10X technology. In contrast, for the meta-analysis across technologies sample-size-based weighting was on average optimal, outperforming standard-error based weighting. These findings suggest that carefully chosen secondary weights can enhance meta-analysis outcomes and guide further method refinement.

## Discussion

Single-cell-derived eQTL studies provide us with the cellular resolution needed to improve our interpretation of disease-associated genetic variants. To be able to capture the full range of genetic variation and gain sufficient statistical power, large sample sizes are required. For this, combining single-cell-derived eQTL summary statistics of many cohorts through meta-analysis is the only strategy that is realistic and able to avoid sharing privacy-sensitive data. However, despite the growth in the number of single-cell-derived eQTL studies in the last 5 years^24–27^, it has remained unclear how best to meta-analyze summary statistics of these studies.

Here we show that an improved weighting scheme can increase the number of eGenes detected without negatively impacting replication rates. We based our WMAs on the weighted Fisher’s method, combined with multiple weights such as sample-size-derived characteristics, and extended this with gene-, SNP-based secondary weights. Although we observed variations in the best-performing weights across different dataset combinations, we can suggest a general list of candidate weights for the optimal WMA performance. In particular, the *average number of cells per donor* and *average number of counts per* cell were consistently top-performing weight for pairwise integration of 10X-derived scRNA-seq datasets. Looking ahead, emerging technologies such as 10x Genomics v4 (gem-X)^28^ and platforms from Parse Biosciences^29^ are likely to introduce additional variability in expression characteristics due to differences in chemistry and capture methods. Although eQTL-scale datasets generated using these newer platforms are not yet publicly available, our framework is well suited to accommodate them once they are. In particular, approaches that incorporate study-specific uncertainty are expected to remain robust across such technological advances. Moreover, the development and adoption of standardized analytical frameworks are critical to improving the generalizability and reproducibility of studies, ensuring that findings remain comparable and robust across diverse datasets and evolving technologies.

When performing pairwise integration of 10X-derived and Smart-seq2 datasets, we observed that *sample size* and *average reads per donor* most often performed best. In contrast, when combining multiple datasets, such as all five PBMC datasets, *standard-error* was the best-performing weight.

This discrepancy between pairwise integration and multi-dataset integration likely reflects the presence of many variable factors across the multiple datasets considered in the meta-analyses, which cannot be easily captured by one specific weight. We expect that therefore the standard error may better capture a combination of these differences, when meta-analyzing multiple datasets whereas the sample-size-like derived parameters may more precisely capture the individual differences and reflect experimental design as a whole when conducting pairwise meta-analysis in specific settings.

To try and address this we explored two alternative approaches to further improve weighting. First, for estimating the performance for the meta-analysis across different technologies we conducted a grid search. We showed that parameter-based weighting of datasets can further improve eGene discovery and replication rate, our grid search analysis showed that none of the individual parameters or parameter combinations we assessed reached the optimum values. While reaching this optimum may remain difficult when using parameter-based weighting, employing a full grid search with multiple datasets is also challenging and not generalizable to subsequent studies. Therefore, we expect that adding even more weights or combining them by methods other than multiplication could further improve parameter-based weighting results, and this should be the way forward.

Second, we also explored directly combining two weights and as a principled alternative. In PBMC and monocyte samples we tested a weighting strategy that adds secondary weights to the overall top-performing primary weight: *average reads per donor*. This analysis showed that *coefficient of variation of single cell expression* and *MAF* is a suitable secondary weight to explore further, as it led to additional improvement in the number of eGenes detected and in the replication rate.

However, still the standard error-based WMA outperforms combined weights in several settings such as meta-analysis of several datasets. So even though it does require substantially more data sharing, sharing both standard error of the actual tests as well as the standard error of the permutations, for an optimal WMA it at least has to be considered.

Finally, although this paper does not focus on the methodology for meta-analyzing overlapping samples, we anticipate that the primary statistical approaches commonly used in bulk data—such as those implemented in the METAL framework—will be adequate for combining single-cell eQTL summary statistics using suggested weights.

## Conclusion

Despite the huge potential of single-cell sequencing technologies, eQTL discovery and summary statistics integration remain complicated due to the low number of samples in individual datasets and technical differences in protocols. Here we provide a means to overcome these difficulties and improve large-scale meta-analysis of sc-eQTL summary statistics, enabling improved power to identify disease-relevant variants without the need to re-analyze data. Here, we present tailored recommendations for conducting meta-analyses on specific dataset combinations, offering adaptable guidelines for integrating single-cell eQTL summary statistics in meta-analytic frameworks. Although we have not covered all possible single-cell technologies, combinations, and dataset-characteristics, we expect that the conclusions drawn from this study are representative for the majority of population-based scRNA-seq data currently available (or to be generated). In particular, we expect that large-scale consortia, in which data-sharing may be difficult and new datasets are continuously added (e.g. sc-eQTLGen^1^ and the Asian Immune Diversity Atlas^30^), will benefit from our recommendations on how to meta-analyze single cell summary statistics. Moreover, we expect that our recommendations reach beyond eQTL meta-analyses as they are broadly applicable to other integrative analyses, such as differential expression analysis or meta-analysis of other biological features and omics layers, in both single-cell and bulk studies.

## Methods

### Single-cell data processing

Processed PBMC scRNA-seq data were obtained from three previously published studies: Wijst et al. (2018)^31^, Oelen et al. (2022)^32^, and Blokland et al. (2023)^33^. We included only unstimulated cells from the Oelen dataset and only cells from 6–8 weeks after myocardial infarction from the patients in the Blokland study to minimize additional variability across datasets due to pathogen stimulation (Oelen study) or effects of the myocardial infarction (Blokland study). For cell type classification, we used the original annotation for the Oelen data and re-annotated the Blokland and Wijst datasets using reference annotation with the Azimuth classification method^34^.

Processed day 0 iPSC data were obtained from the Cuomo et al. study^22^. To only meta-analyze independent samples, we split the 87 iPSC samples into three sample sets (Sets A, B, C, and D): 25 samples with 10X data (Set C), 25 samples with Smart-Seq2 data (Set B), 62 non-overlapping Smart-Seq2 samples (Set A), and 25 random samples from Set A (Set D).

## sc-eQTL mapping

To conduct *cis*-eQTL mapping in both PBMC and iPSC samples, we utilized a methodology outlined by Cuomo et al.^22^. Our eQTL approach only considered common variants (MAF > 10% and Hardy-Weinberg equilibrium P < 0.001) within 100 kb upstream and downstream of the gene body. These variants were linked to potential target genes within a 100 kb window using linear mixed models fitted with LIMIX (version: 2.0.3)^35^. We quantile-normalized the expression profile of each gene to a standard normal distribution and used the Plink identity-by-descent matrix to correct for population stratification as a random effect. Per dataset, we performed 1,000 permutations while estimating the number of independent loci tested and calibrating each association per gene by fitting a beta-distribution over the permutations, as outlined in QtlTools^36^. To standardize the multiple testing correction across the single-cell studies, we took the alpha and beta parameters per gene from the beta-distribution from the largest study, per cellular context (i.e. iPSCs, PBMCs, or monocytes), and used this to correct the smaller studies. This standardization was done to overcome the differences in permutations. Lastly, we matched the dataset summary statistics of the individual datasets to the effect alleles of the reference (bulk) dataset: eQTLGen for PBMC and monocytes and HipSci for the iPSC data.

## Meta-analysis

The weighted Fisher’s method is a statistical technique used to combine the results of multiple independent statistical tests into a single summary statistic. It assigns weights to an individual Z-score based on some criteria (e.g. the sample size or the reliability of each test), after which Z-scores over multiple studies are combined to calculate an overall statistic that assesses the significance of the combined evidence (Eq. 1).

1 $$Z_{w} = \frac{{\sum\nolimits_{i = 1}^{k} {w_{i} } Z_{i} }}{{\sqrt {\sum\nolimits_{i = 1}^{k} {w_{i}^{2} } } }}$$

where w_i_ = specific weight or a weights combination, Z_i_ = Z-score, and i = number of the effect.

## Testing of WMA

The meta-analysis of PBMC samples was conducted utilizing two approaches: pairwise meta-analysis and integration of all five datasets simultaneously. The former approach enabled assessment of the uniformity of the weighting strategies employed for datasets with different chemistries and sample sizes. The integration of all the datasets, which represents the conventional mode of meta-analysis, was implemented to find the overall optimal weighting scheme. For the iPSC data, we could only assess the integration of two datasets.

## Ranking and definition of precision and sensitivity

We used an adapted F1 score, named F1*, to find the optimal WMA strategy because it considers both precision and recall, providing a balanced measure of the model’s ability to correctly classify positive samples while minimizing false positives and false negatives. This F1* score follows the same formula as the original F1 score:

$${\text{F1}}\;{\text{Score}} = \frac{TP}{{TP + \frac{1}{2}(FP + FN)}}$$

where TP = the proportion of true positives, FN = the proportion of false negatives and FP = the proportion of false positives. For F1* we changed the TP and FN definitions to only include eQTL effects that were ever identified in the single cell data and bulk eQTL summary statistics. To this end, a positive (TP+FN) effect is one that is detected in any of the weighted meta-analyses and the reference sample (Supplementary Figure 5).

After calculating the F1* score using the new reference set, we calculated a weighted mean of F1* between the to-be-integrated datasets using the *total number of samples* in the meta-analysis as a weight. This mean was used to find the best-performing WMA approach for different combinations of the datasets, for instance to determine the best-performing WMA approach to integrate summary statistics of 10X and Smart-seq2 samples in iPSC samples or that between datasets with different version chemistries in PBMC samples.

## Grid search

To assess whether the integration of the 10X and Smart-seq2 samples could be improved, we systematically varied the weight assigned to one dataset while keeping the weight of the other dataset constant at 1 using a grid search. The varying weight was adjusted incrementally from 0.1 to 10, spanning a range of values that were similar but broader than the assessed value in the summary-statistic-based meta-analysis. Following this weight adjustment, the results of the grid search were visualized by applying a log10-transformation to the changing weight values.

## Supplementary Information

Supplementary Information 1.

Supplementary Information 2.

Supplementary Information 3.

Supplementary Information 4.

Supplementary Information 5.

Supplementary Information 6.

## Data Availability

Code is available on Github: https://github.com/sc-eQTLgen-consortium/sc_wma. Input files are available at Zenodo: https://zenodo.org/records/11,126,637.

## Abbreviations

10X: 10X genomics scRNA-sequencing
(ct)-eQTL: (Cell-type-specific) expression quantitative trait locus
GWAS: Genome-wide association study
eGene: Gene that has one or more eQTLs
FDR: False discovery rate
MAF: Minor allele frequency
MTC: Multiple testing correction
PBMC: Peripheral blood mononuclear cell
scRNA-seq: Single-cell RNA-sequencing
WMA: Weighted meta-analysis

## References

[1] Ashton, J. M. *et al*. Comparative analysis of single-Cell RNA sequencing platforms and methods. *J. Biomol. Tech. JBT* **32**, 3fc1f5fe.3eccea01 (2021). 10.7171/3fc1f5fe.3eccea01 PMC9258609 35837267

[2] Gezelius, H. *et al*. Comparison of high-throughput single-cell RNA-seq methods for ex vivo drug screening. *NAR Genom. Bioinforma.* **6**, lqae001 (2024). 10.1093/nargab/lqae001 PMC10823582 38288374

[3] Mereu, E. *et al*. Benchmarking single-cell RNA-sequencing protocols for cell atlas projects. *Nat. Biotechnol.* **38**, 747–755 (2020). 32518403 10.1038/s41587-020-0469-4

[4] Svensson, V., Vento-Tormo, R. & Teichmann, S. A. Exponential scaling of single-cell RNA-seq in the past decade. *Nat. Protoc.* **13**, 599–604 (2018). 29494575 10.1038/nprot.2017.149

[5] van der Wijst, M. *et al*. The single-cell eQTLGen consortium. *Elife* **9**, e52155 (2020). 32149610 10.7554/eLife.52155 PMC7077978

[6] The single-cell eQTLGen consortium | eLife. https://elifesciences.org/articles/52155v1. 10.7554/eLife.52155 PMC7077978 32149610

[7] Yazar, S. *et al*. Single-cell eQTL mapping identifies cell type–specific genetic control of autoimmune disease. *Science* **376**, eabf3041 (2022). 35389779 10.1126/science.abf3041

[8] A mega-analysis of expression quantitative trait loci (eQTL) provides insight into the regulatory architecture of gene expression variation in liver | Scientific Reports. https://www.nature.com/articles/s41598-018-24219-z. 10.1038/s41598-018-24219-z PMC5897392 29650998

[9] Zaykin, D. V. Optimally weighted Z-test is a powerful method for combining probabilities in meta-analysis. *J. Evol. Biol.* **24**, 1836–1841 (2011). 21605215 10.1111/j.1420-9101.2011.02297.x PMC3135688

[10] Willer, C. J., Li, Y. & Abecasis, G. R. METAL: fast and efficient meta-analysis of genomewide association scans. *Bioinformatics* **26**, 2190–2191 (2010). 20616382 10.1093/bioinformatics/btq340 PMC2922887

[11] Chen, Z. Is the weighted z-test the best method for combining probabilities from independent tests?. *J. Evol. Biol.* **24**, 926–930 (2011). 21401770 10.1111/j.1420-9101.2010.02226.x

[12] Whitlock, M. C. Combining probability from independent tests: the weighted Z-method is superior to Fisher’s approach. *J. Evol. Biol.* **18**, 1368–1373 (2005). 16135132 10.1111/j.1420-9101.2005.00917.x

[13] Li, Y. & Ghosh, D. Meta-analysis based on weighted ordered P-values for genomic data with heterogeneity. *BMC Bioinform.* **15**, 226 (2014). 10.1186/1471-2105-15-226 PMC4089554 24972803

[14] Laoutidis, Z. G. & Luckhaus, C. The Liptak-Stouffer test for meta-analyses. *Biol. Psychiatry* **77**, e1–e2 (2015). 24655596 10.1016/j.biopsych.2013.11.033

[15] Yoon, S., Baik, B., Park, T. & Nam, D. Powerful p-value combination methods to detect incomplete association. *Sci. Rep.* **11**, 6980 (2021). 33772054 10.1038/s41598-021-86465-y PMC7997958

[16] Borenstein, M., Hedges, L. V., Higgins, J. P. T. & Rothstein, H. R. A basic introduction to fixed-effect and random-effects models for meta-analysis. *Res. Synth. Methods* **1**, 97–111 (2010). 26061376 10.1002/jrsm.12

[17] Full-length RNA-seq from single cells using Smart-seq2 | Nature Protocols. https://www.nature.com/articles/nprot.2014.006. 10.1038/nprot.2014.006 24385147

[18] Chromium Single Cell 3’ v2 Libraries – Sequencing Metrics for Illumina® Sequencers. *10x Genomics* https://www.10xgenomics.com/support/universal-three-prime-gene-expression/documentation/steps/sequencing/chromium-single-cell-3-v-2-libraries-sequencing-metrics-for-illumina-r-sequencers.

[19] Chromium Single Cell 3’ Reagent Kits User Guide (v3.1 Chemistry) - 10x Genomics. https://www.10xgenomics.com/support/universal-three-prime-gene-expression/documentation/steps/library-prep/chromium-single-cell-3-reagent-kits-user-guide-v-3-1-chemistry.

[20] The Human Cell Atlas - PubMed. https://pubmed.ncbi.nlm.nih.gov/29206104/.

[21] Large-scale cis- and trans-eQTL analyses identify thousands of genetic loci and polygenic scores that regulate blood gene expression | Nature Genetics. https://www.nature.com/articles/s41588-021-00913-z. 10.1038/s41588-021-00913-z PMC8432599 34475573

[22] Cuomo, A. S. E. *et al*. Single-cell RNA-sequencing of differentiating iPS cells reveals dynamic genetic effects on gene expression. *Nat. Commun.* **11**, 810 (2020). 32041960 10.1038/s41467-020-14457-z PMC7010688

[23] Identification of rare and common regulatory variants in pluripotent cells using population-scale transcriptomics | Nature Genetics. https://www.nature.com/articles/s41588-021-00800-7. 10.1038/s41588-021-00800-7 PMC7944648 33664507

[24] Single-cell RNA-seq reveals cell type-specific molecular and genetic associations to lupus - PubMed. https://pubmed.ncbi.nlm.nih.gov/35389781/. 10.1126/science.abf1970 PMC9297655 35389781

[25] Single-cell eQTL mapping identifies cell type–specific genetic control of autoimmune disease | Science. https://www.science.org/doi/10.1126/science.abf3041. 10.1126/science.abf3041 35389779

[26] AIDA - Overview - HCA Data Explorer. https://explore.data.humancellatlas.org/projects/f0f89c14-7460-4bab-9d42-22228a91f185.

[27] Integrated analysis of multimodal single-cell data: Cell. https://www.cell.com/cell/fulltext/S0092-8674(21)00583-3. 10.1016/j.cell.2021.04.048 PMC8238499 34062119

[28] Ortolano, N. The neXt generation of single cell RNA-seq: An introduction to GEM-X technology. *10x Genomics* https://www.10xgenomics.com/blog/the-next-generation-of-single-cell-rna-seq-an-introduction-to-gem-x-technology.

[29] Tran, V. *et al.* High sensitivity single cell RNA sequencing with split pool barcoding. 2022.08.27.505512 Preprint at 10.1101/2022.08.27.505512 (2022).

[30] Kock, K. H. *et al*. Asian diversity in human immune cells. *Cell* **188**, 2288-2306.e24 (2025). 40112801 10.1016/j.cell.2025.02.017

[31] van der Wijst, M. G. P. *et al*. Single-cell RNA sequencing identifies celltype-specific cis-eQTLs and co-expression QTLs. *Nat. Genet.* **50**, 493–497 (2018). 29610479 10.1038/s41588-018-0089-9 PMC5905669

[32] Oelen, R. *et al*. Single-cell RNA-sequencing of peripheral blood mononuclear cells reveals widespread, context-specific gene expression regulation upon pathogenic exposure. *Nat. Commun.* **13**, 3267 (2022). 35672358 10.1038/s41467-022-30893-5 PMC9174272

[33] van Blokland, I. V. *et al*. Single-cell dissection of the immune response after acute myocardial infarction. *Circ. Genomic Precis. Med.* **17**, e004374 (2024). 10.1161/CIRCGEN.123.004374 PMC11188632 38752343

[34] Hao, Y. *et al*. Integrated analysis of multimodal single-cell data. *Cell* **184**, 3573-3587.e29 (2021). 34062119 10.1016/j.cell.2021.04.048 PMC8238499

[35] Limix’s documentation—limix 2.0.0a3 documentation. https://horta-limix.readthedocs.io/en/api/index.html.

[36] A complete tool set for molecular QTL discovery and analysis | Nature Communications. https://www.nature.com/articles/ncomms15452. 10.1038/ncomms15452 PMC5454369 28516912

