# Supplementary material for: Optimized summary-statistic-based single-cell eQTL meta-analysis
Source: Sci Rep. 2025 Aug 4;15:28407. doi: 10.1038/s41598-025-08808-3 (PMC12322206; doi:10.1038/s41598-025-08808-3)

## Supplemental Material

### Supplementary Tables

**Table S1**: Dataset Sample-size-like characteristics. a) 10X Pseudobulk samples; b) 10X Monocyte samples; c) iPSC samples

**Table S2**: WMA of sample-size-like weights using eQTLGen reference. a) 10X Pseudobulk samples; b) 10X Monocyte samples; c) iPSC samples

**Table S3**: Grid search results in iPSC samples. a) 10X and Smart-seq2 same-size meta-analysis; b) 10X and Smart-seq2 different-size meta-analysis.

**Table S4**: WMA of the best performing sample-size-like weight and auxiliary weights. a) 10X Pseudobulk samples (sample-size weight: average number of cells per donor); b) 10X Monocyte samples (sample-size weight: average number of cells per donor); c) iPSC samples (sample-size weight: number of donors)

**Table S5**: WMA of all weights. a) 10X Pseudobulk samples; b) 10X Monocyte samples; c) iPSC samples

###

### Supplementary figures

**Figure S1. 10X sequencing expression patterns (PBMC and Monocytes).** Distribution of the number of cells (a), number of counts per sample (b) and counts per cell (c) in pbmc samples; proportion of the cell types in the datasets (d); distribution of mean expression (e) and standard deviation (f) per dataset in pbmc (green) and monocyte (blue) samples; correlation of expression-like parameters (weights) between pbmc and monocyte samples (g).


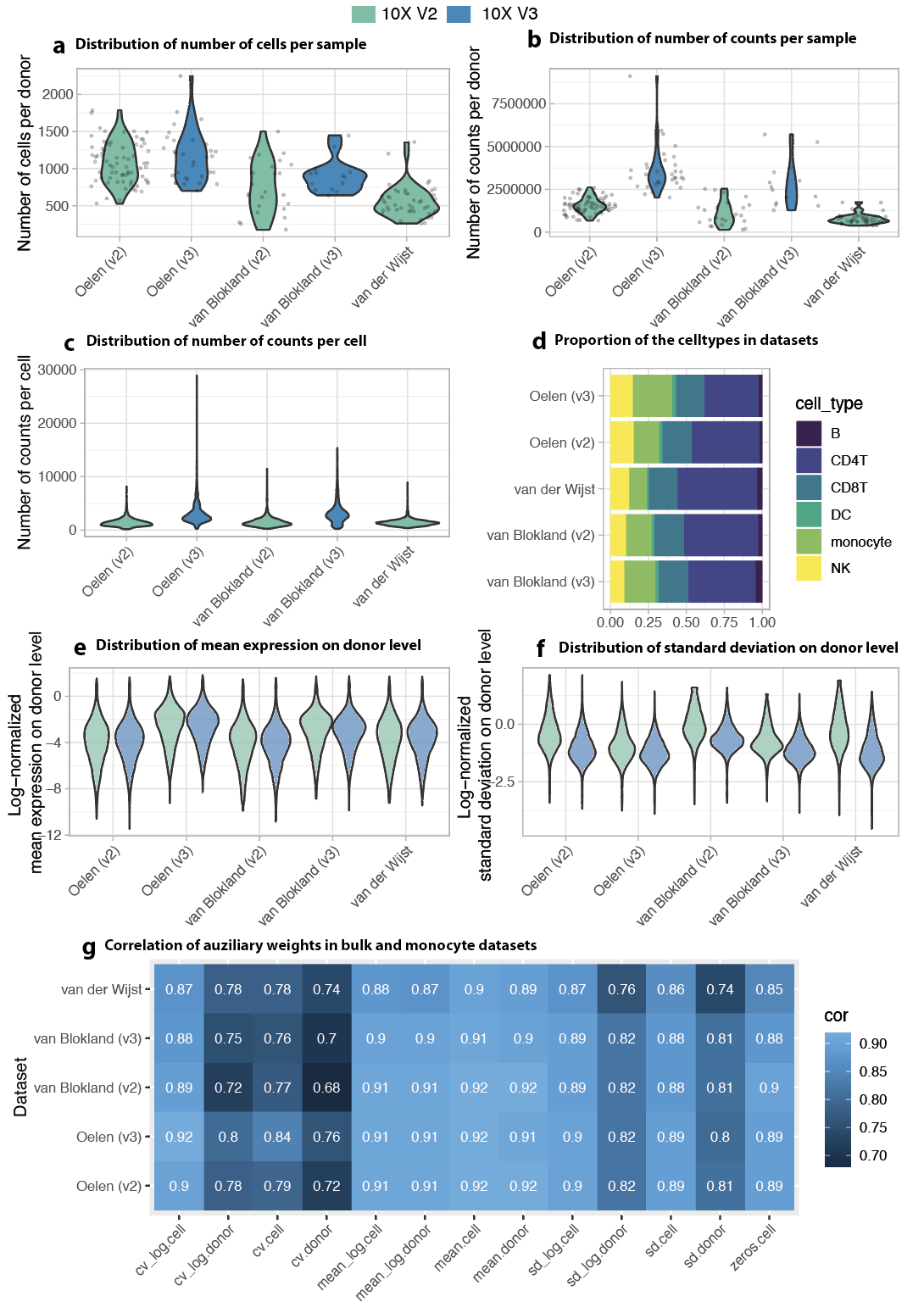


**Figure S2. Smart-seq2 & 10X expression patterns (iPSC).** Distribution of the number of cells (a), number of counts per sample (b) and counts per cell (c) in pbmc samples; distribution of mean expression (d), standard deviation (e) and coefficient of variation (f) per dataset in 10X (green) and Smart-seq2 (blue) samples.


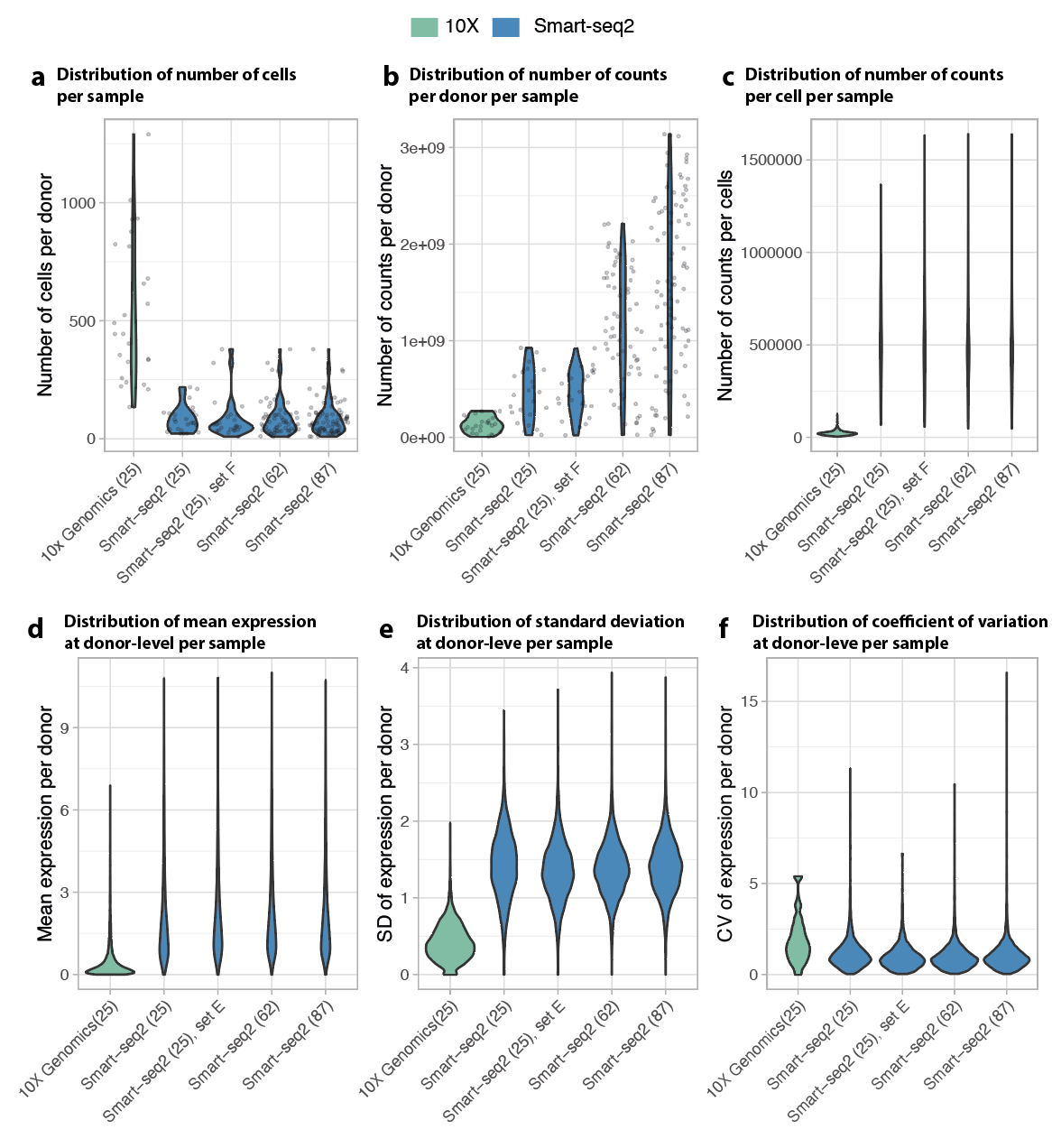


**Figure S3. eQTL mapping in original samples.** a) Overlap of significant eQTLs in PBMC dataset; b) Correlation of all eQTL effects Z-score in PBMC dataset with eQTLGen Z-score (reference); c) Overlap of significant eQTLs in monocyte dataset; d) Correlation of all eQTL effects Z-score in monocyte dataset with eQTLGen Z-score (reference); e) Overlap of significant eQTLs in iPSC dataset; f) Correlation of all eQTL effects in iPSC dataset with Z-score from the bulk sample Z-score (reference);
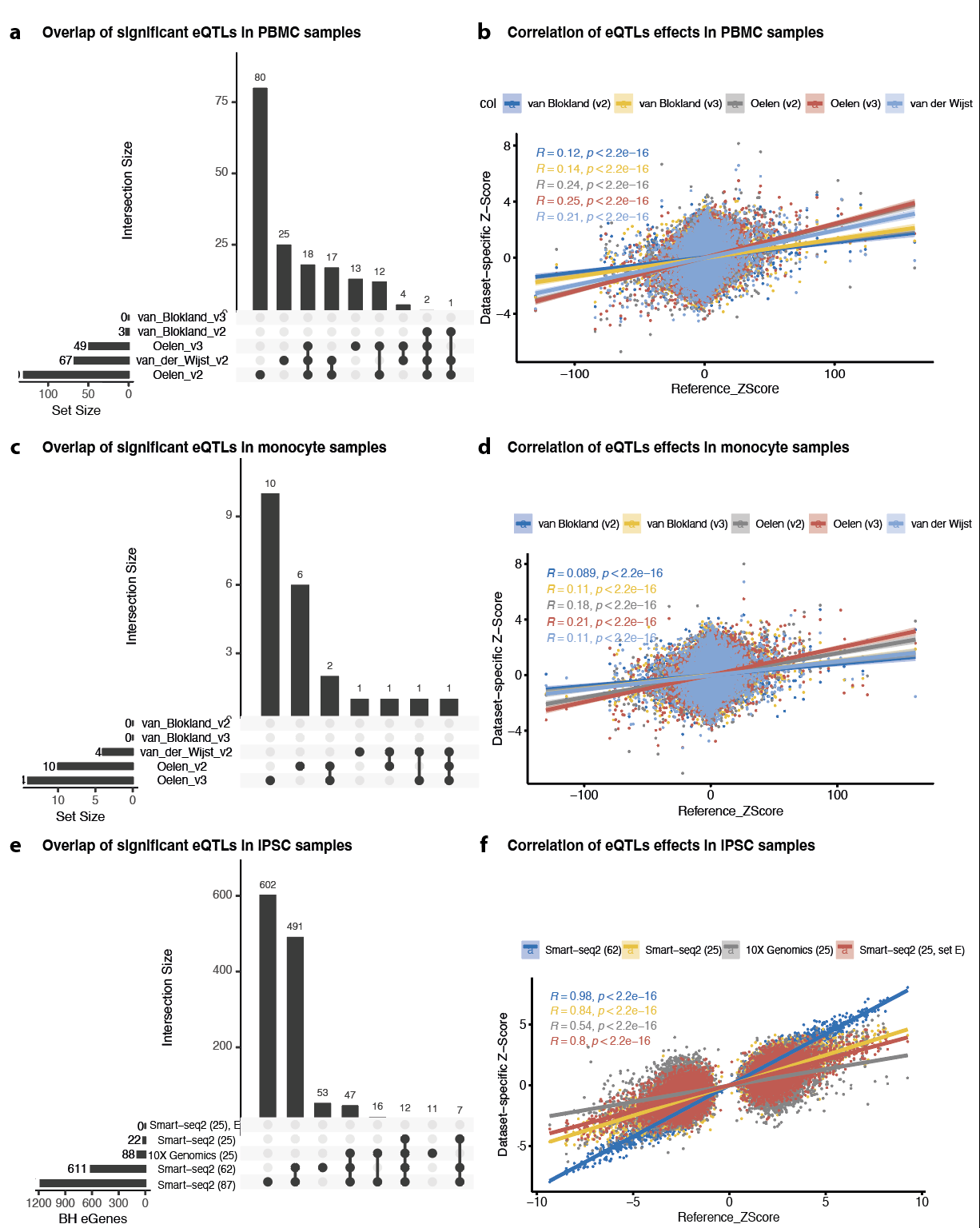


**Figure S4. Weighted meta-analyses with the redefined reference sample (OneK1K).** Number of datasets where using a secondary weight shows an improvement over sample-size based method in eQTLGen (a) and OneK1K references (b); change in number of genes detected over Average number of cells when using a combination of Average number of cells with a secondary weight (c ) in OneK1K.

**
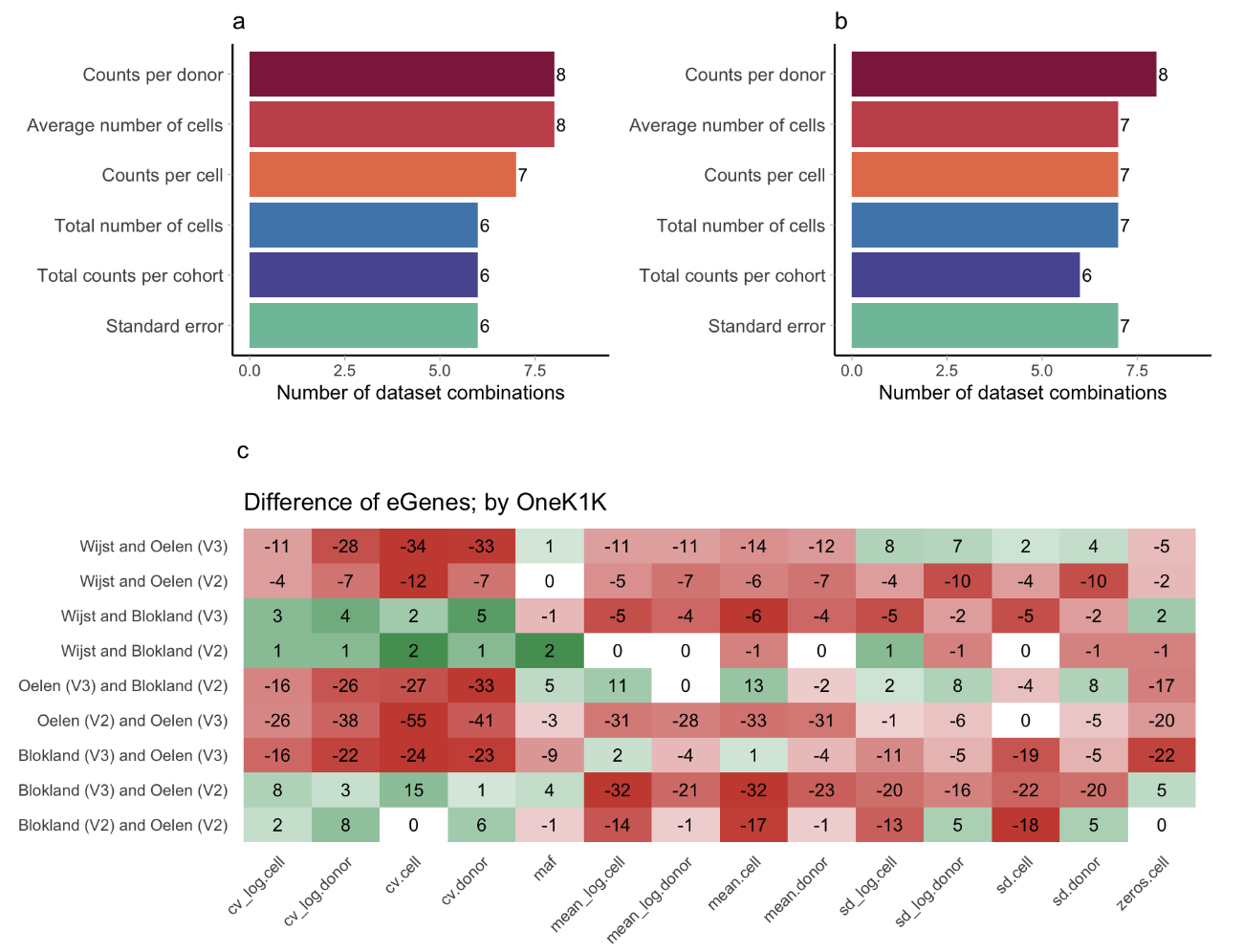
**

**Figure S5. Weighted meta-analyses with the redefined reference sample.** The full description is available in Methods (Section 5).


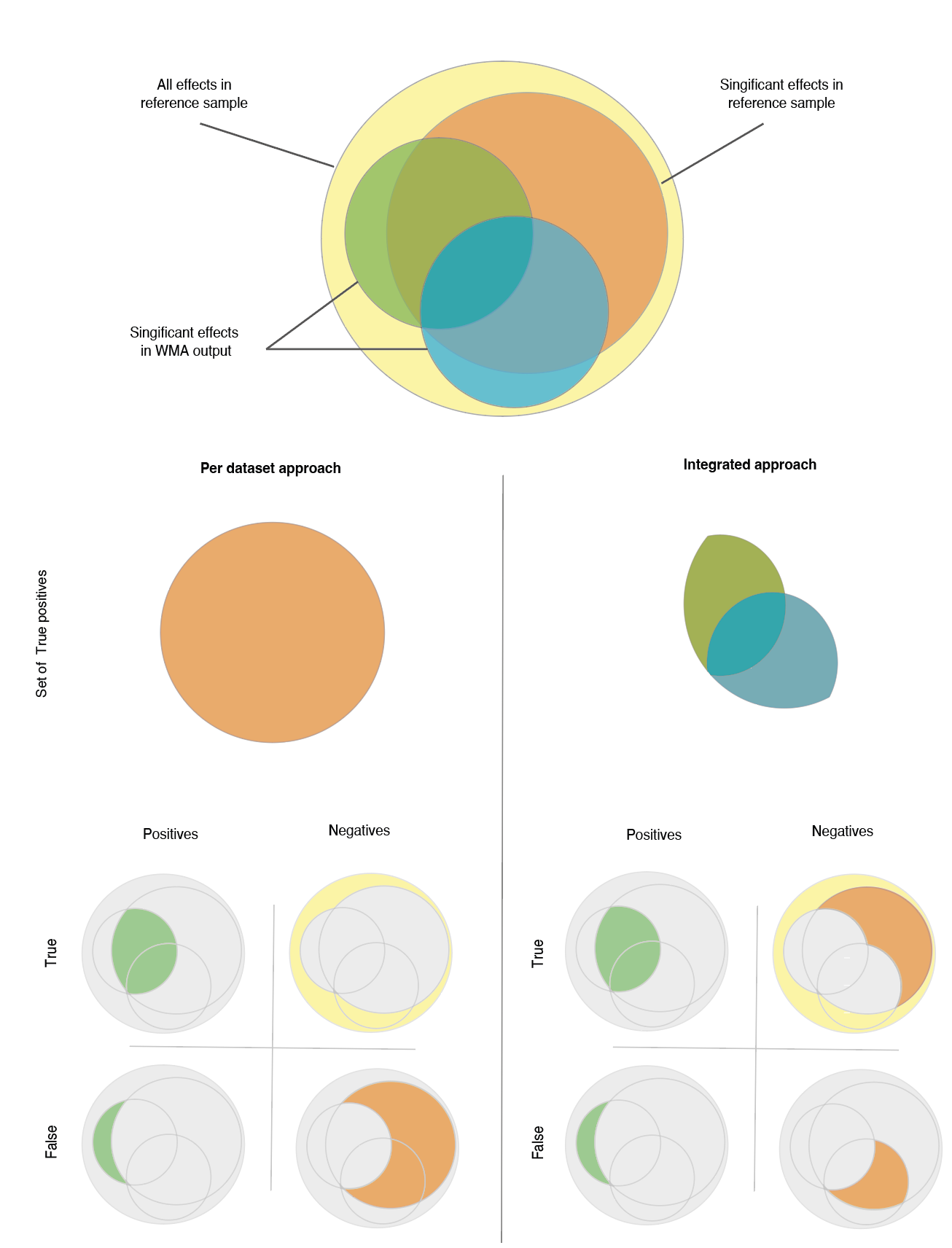

Supplement: Supplementary file 1 — Supplementary Information 1. [file 41598_2025_8808_MOESM1_ESM.docx]
